# Bioinformatics and system biology approaches to determine the connection of SARS-CoV-2 infection and intrahepatic cholangiocarcinoma

**DOI:** 10.1371/journal.pone.0300441

**Published:** 2024-04-22

**Authors:** Xinyi Zhou, Tengda Huang, Hongyuan Pan, Ao Du, Tian Wu, Jiang Lan, Yujia Song, Yue Lv, Fang He, Kefei Yuan

**Affiliations:** 1 Division of Liver Surgery, Department of General Surgery and Laboratory of Liver Surgery, and State Key Laboratory of Biotherapy, West China Hospital, Sichuan University, Chengdu, China; 2 Center of Infectious Diseases, West China Hospital, Sichuan University, Chengdu, China; 3 NHC Key Laboratory of Transplant Engineering and Immunology, Regenerative Medicine Research Center, Frontiers Science Center for Disease-related Molecular Network, West China Hospital of Sichuan University, Chengdu, China; Concordia University, CANADA

## Abstract

**Introduction:**

Severe acute respiratory syndrome coronavirus 2 (SARS-CoV-2), the causal agent of coronavirus disease 2019 (COVID-19), has infected millions of individuals worldwide, which poses a severe threat to human health. COVID-19 is a systemic ailment affecting various tissues and organs, including the lungs and liver. Intrahepatic cholangiocarcinoma (ICC) is one of the most common liver cancer, and cancer patients are particularly at high risk of SARS-CoV-2 infection. Nonetheless, few studies have investigated the impact of COVID-19 on ICC patients.

**Methods:**

With the methods of systems biology and bioinformatics, this study explored the link between COVID-19 and ICC, and searched for potential therapeutic drugs.

**Results:**

This study identified a total of 70 common differentially expressed genes (DEGs) shared by both diseases, shedding light on their shared functionalities. Enrichment analysis pinpointed metabolism and immunity as the primary areas influenced by these common genes. Subsequently, through protein-protein interaction (PPI) network analysis, we identified *SCD*, *ACSL5*, *ACAT2*, *HSD17B4*, *ALDOA*, *ACSS1*, *ACADSB*, *CYP51A1*, *PSAT1*, and *HKDC1* as hub genes. Additionally, 44 transcription factors (TFs) and 112 microRNAs (miRNAs) were forecasted to regulate the hub genes. Most importantly, several drug candidates (Periodate-oxidized adenosine, Desipramine, Quercetin, Perfluoroheptanoic acid, Tetrandrine, Pentadecafluorooctanoic acid, Benzo[a]pyrene, SARIN, Dorzolamide, 8-Bromo-cAMP) may prove effective in treating ICC and COVID-19.

**Conclusion:**

This study is expected to provide valuable references and potential drugs for future research and treatment of COVID-19 and ICC.

## 1. Introduction

Coronavirus disease 2019 (COVID-19), first identified in December 2019 [1], is a recently discovered respiratory ailment caused by the severe acute respiratory syndrome coronavirus 2 (SARS-CoV-2). While most COVID-19 patients experience mild to moderate symptoms, 5% suffer from acute respiratory distress syndrome (ARDS), multiple organ failure, or septic shock, and approximately 15% develop severe pneumonia [2]. New SARS-CoV-2 variants, such as Alpha, Beta, Delta, and Omicron, continue to emerge, leading to high case rates and significant global mortality. As of November 2023, the World Health Organization (WHO) has reported 772,052,752 COVID-19 cases, resulting in 6,985,278 deaths [3]. Previous research indicates that SARS-CoV-2 primarily infects human cells when its surface spike protein binds to the angiotensin-converting enzyme 2 (ACE2) receptor [4]. The spike protein is the protein for SARS-CoV-2 to recognize host cells and is also the main target of the human immune system [5].

Although the virus directly infects the lungs, its effect on the liver cannot be ignored. Patients with severe COVID-19 seem to have higher rates of liver dysfunction [6]. Patients with liver dysfunction are identified to have high risk of developing severe COVID-19 [7, 8]. Clinical data also demonstrate that patients with comorbidities fare worse than those without them [9, 10]. Besides, cancer patients undergoing chemotherapy or immunotherapy are more susceptible to COVID-19 infection [11]. Bioinformatics approaches have been employed to investigate the link between colorectal cancer and COVID-19 [12]. Liver cancer, ranking as the sixth most common and third deadliest malignancy globally [13], is closely associated with cirrhosis, Hepatitis B Virus (HBV) and Hepatitis C Virus (HCV) infections, and metabolic syndrome [14]. First-line (gemcitabine and cisplatin), second-line (FOLFOX), and adjuvant (capecitabine) systemic chemotherapy are currently the accepted standard of treatment of ICC [15]. In addition, ICC patients often experience severe liver dysfunction. Therefore, to better overcome COVID-19 and ICC in the future, it is imperative to explore and clarify the internal molecular mechanism between these two diseases.

**Fig 1:**
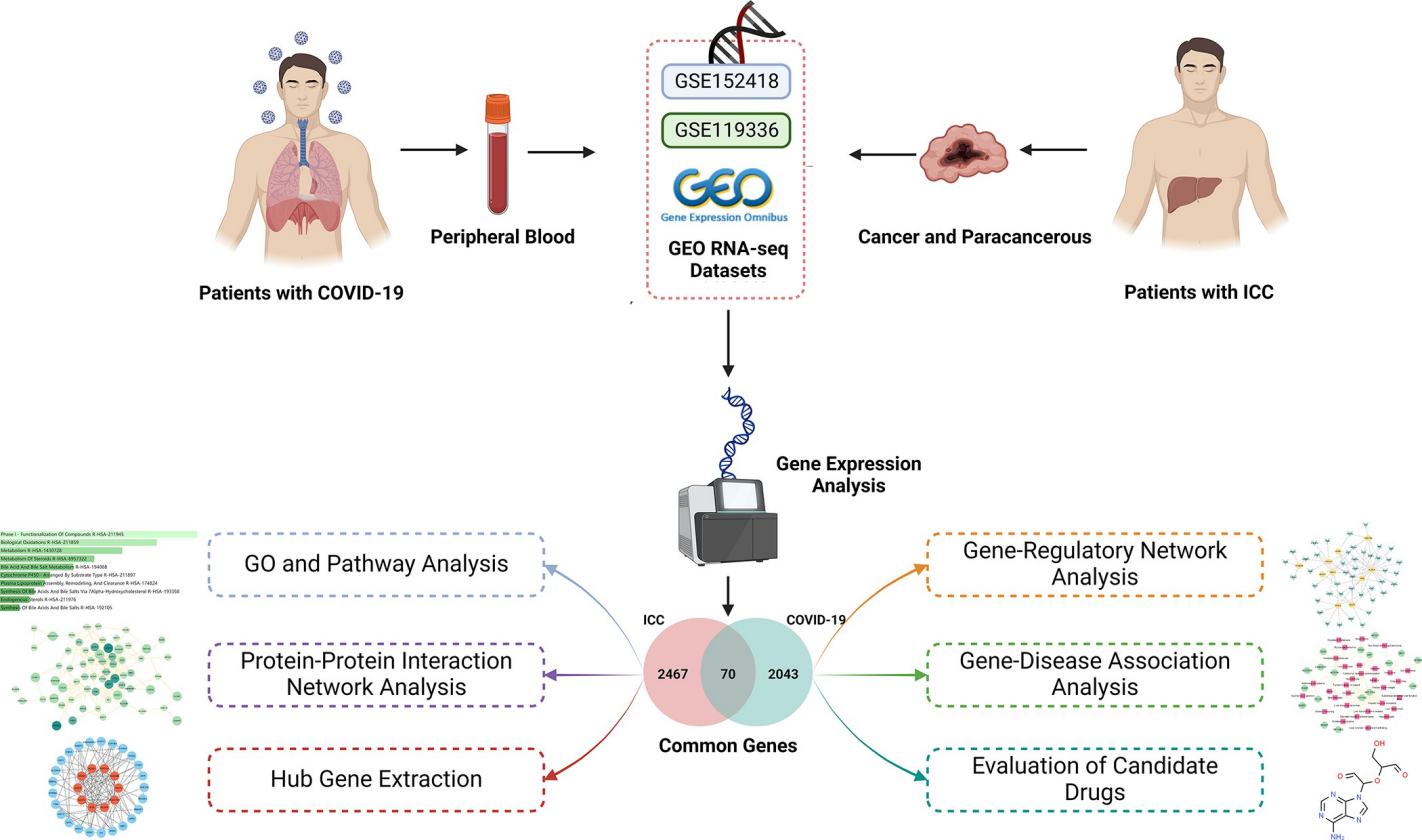
Schematic illustration of the overall general workflow of this study.

This study utilized two datasets, GSE152418 and GSE119336, obtained from the Gene Expression Omnibus (GEO) database, to investigate the correlations between ICC and COVID-19. Differentially expressed genes (DEGs) were identified, and then 70 shared DEGs genes were found for both diseases. Pathway analysis was conducted using these mutual DEGs to gain insights into the underlying gene expression mechanisms. To gather hub genes, a protein-protein interaction (PPI) network was constructed using the 70 recognized DEGs. Next, the hub genes were used to elucidate the gene-regulatory network, predict potential drugs, and complete the gene-disease association network. A flowchart of the overall work is presented in Fig 1. The findings of this study will enhance our understanding of the interplay between COVID-19 and ICC, aid in drug selection, and facilitate the development of novel therapeutic strategies for combatting both diseases.

## 2. Materials and methods

### 2.1 Collection of gene expression datasets

RNA-seq datasets from the NCBI [16], GEO (http://www.ncbi.nlm.nih.gov/geo) database were obtained to investigate common biological interrelationships between COVID-19 and ICC. The COVID-19 dataset, with GEO accession ID GSE152418, comprises transcriptional profiles from 17 healthy individuals and 17 COVID-19-infected individuals, sequenced using the Illumina NovaSeq 6000 platform (Homo sapiens) for RNA extraction [17]. The ICC dataset (GEO accession ID: GSE119336) contains 15 pairs of human ICC tumors and non-tumor liver tissues that were sequenced using an Illumina HiSeq 2000 (Homo sapiens) high-throughput sequencing donated by Zhang et al. [18].

### 2.2 Identification of differentially expressed genes and shared differentially expressed genes between ICC and COVID-19

DEGs are genes that exhibit significant differences in transcriptional levels among various test conditions [19]. The DEGs of GSE119336 and GSE152418 were identified from the expression values by R (version 4.2.1) software with the LIMMA package [20] and corrected by Benjamini-Hochberg to reduce the error detection rate (FDR). A cutoff criterion of FDR < 0.05 and |log_2_ Fold Change| > 1 was employed to identify significant DEGs in both datasets. The shared DEGs of GSE119336 and GSE152418 were acquired using the jvenn [21] (http://jvenn.toulouse.inra.fr/app/example.html), an online VENN graph mapping platform, to plot VENN analysis.

### 2.3 Gene ontology and pathway enrichment analysis

Gene enrichment analysis is a crucial analytical approach for categorizing genes into biological functions [22]. In order to understand the function of common DEGs, we performed gene ontology (GO) and pathway enrichment analyses connected with the mutual DEGs using Enrichr [23] (https://maayanlab.cloud/Enrichr/), a wide range of online gene set enrichment tool. The three types of GO database in the GO database are biological processes (BP), molecular functions (MF), and cellular components (CC). In pathway enrichment analysis, four databases were regarded, including the Kyoto Encyclopedia of Genes and Genomes (KEGG), Wikipathways, Reactome, and the Bioplanet. The P-value < 0.05 was used as a criterion to screen for reliable results.

### 2.4 Protein-protein interaction network analysis and hub gene extraction

The PPI was established using the STRING [24] (version 11.5) database (https://cn.string-db.org/), an online protein-protein association networks platform, and was then visualized and drawn as a network using Cytoscape [25] (version 3.9.1), an open source software platform for visualizing complex networks. A PPI network was constructed based on proteins encoded by common DEGs shared between COVID-19 and ICC, using a composite score threshold of 0.15. Hub genes, which demonstrate strong connections within potential modules [26], were predicted using the Cytoscape plug-in cytoHubba.

### 2.5 Gene-regulatory network analysis

To discover the transcriptional factors (TFs) and microRNAs (miRNAs) that regulate the hub genes post-transcriptionally, hub gene-TF interplay networks and hub gene-miRNA interaction networks have been dug by means of NetworkAnalyst [27] (version 3.0), a comprehensive visual analysis platform for gene expression profiling. The hub gene-TF interaction networks were built according to the JASPAR [28] database. Hub gene-miRNA interaction networks were constructed via the TarBase [29] (version 8.0) databases.

### 2.6 Gene-disease association analysis

In order to study the human genetic illnesses of shared genes between COVID-19 and ICC, DisGeNET [30] (https://www.disgenet.org/), a publicly accessible database containing information on approximately 24,000 diseases, 17,000 genes, and 117,000 genetic variations related to human illnesses, was used in our analysis. Similarly, NetworkAnalyst and Cytoscape were used to dig gene-disease relationships in order to find diseases associated with common DEGs.

### 2.7 Evaluation of candidate drugs

To anticipate protein-drug interactions and discover prospective pharmacological substances related to hub genes, we employed the Drug Signatures Database [31] (DSigDB, http://tanlab.ucdenver.edu/DSigDB), which contains 17,389 unique chemicals that span 19,531 genes and has 22,527 gene sets. Using the Enrichr web server and the DSigDB database, medicines targeting hub genes were identified between the COVID-19 and ICC datasets using a statistical threshold of P-value < 0.05.

## 3. Results

### 3.1 Recognition of differentially expressed genes and biological relationships between ICC and COVID-19

**Fig 2:**
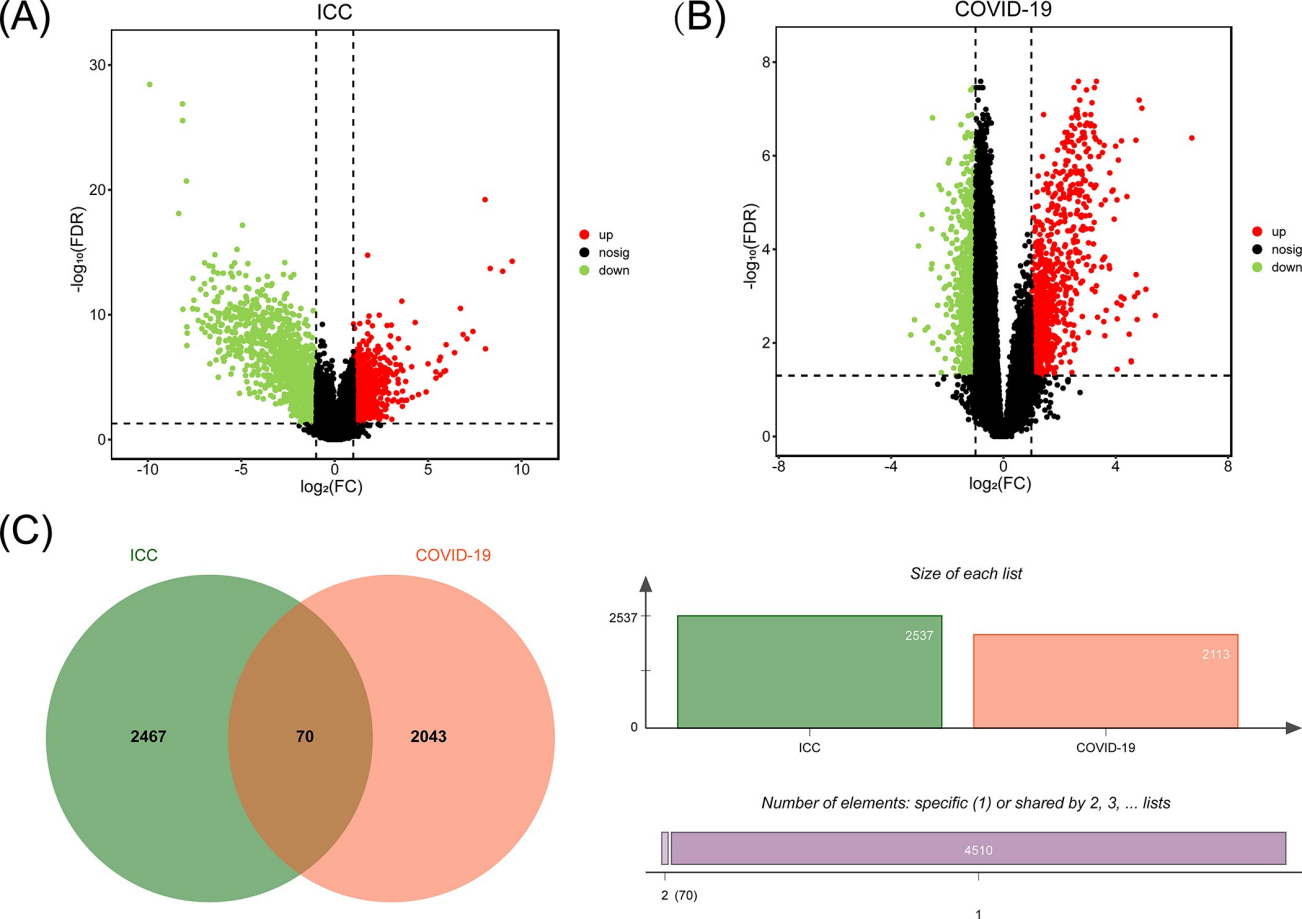
Volcano plots and Venn diagram depicts the shared DEGs among COVID-19 and ICC. Volcano plots of (A) COVID-19 and (B) ICC, with genes with |log2Fold Change| > 1 and FDR < 0.05. (C) The Venn diagram depicts the shared DEGs among COVID-19 and ICC.

In order to evaluate the interactions and implications of ICC with COVID-19, the RNA-seq dataset was examined from the National Center for Biotechnology Information (NCBI). In the ICC dataset, 2,537 DEGs was found, including 1,095 were up-regulated and 1,442 were down-regulated (Fig 2A and S1 Table). Meanwhile, there are 1,267 up-regulated and 891 down-regulated genes showed in the COVID-19 dataset (Fig 2B and S2 Table). Table 1 is a list of the condensed data of DEGs. With the use of the cross-comparative analysis, we were able to find 70 DEGs that were shared by the ICC and COVID-19 datasets (Fig 2C and S3 Table). These outcomes revealed that COVID-19 and ICC had certain molecular similarities.

**Table 1:** Overview of datasets with their geo-features and their quantitative measurements in this analysis.

Disease name	GEO accession	GEO platform	Total DEGs count	Up regulated DEGs count	Down regulated DEGs count
ICC	GSE119336	GPL11154	2,537	1,095	1,442
COVID-19	GSE152418	GPL24676	2,113	1,267	846

### 3.2 Gene enrichment analyses of shared DEGs

Our study used gene ontology and pathway enrichment analysis to learn more about these typical DEGs’ roles and signaling pathways. Gene functional similarity is frequently assessed using the GO enrichment analysis [32]. A modeling technique called pathway analysis is used to show how crucial molecular or biological processes interact and illustrate the reciprocal impacts of various diseases [33]. In order to uncover highly enriched functional GO keywords and pathways, we ran a functional-enrichment test on common DEGs using the Enrichr program.

**Fig 3:**
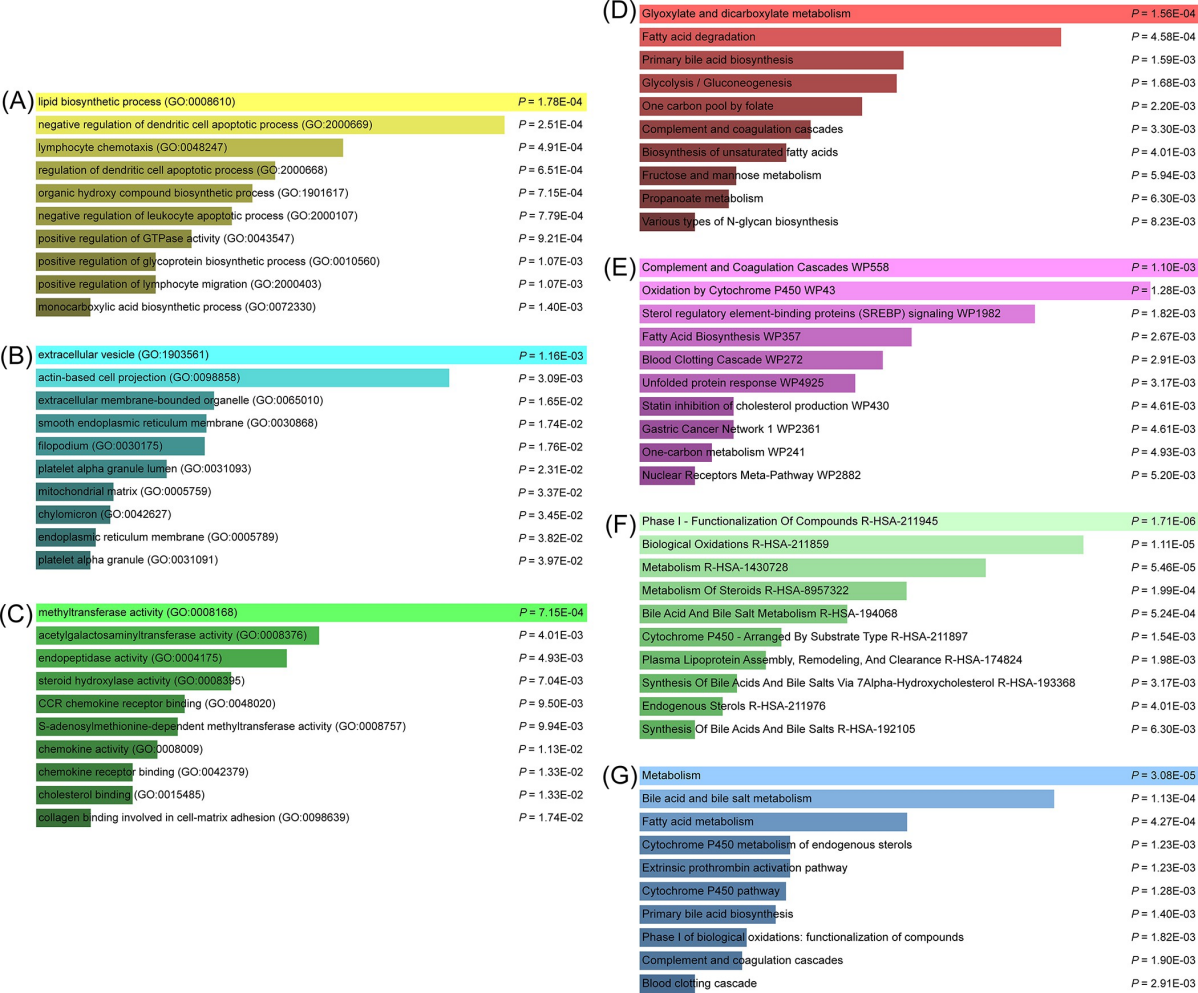
Analysis of common DEGs between COVID-19 and ICC using ontology and pathway enrichment. Ontological analysis: (A) Biological processes, (B) Molecular function, and (C) Cellular components. Pathway enrichment analysis: (D) KEGG, (E) Wikipathways, (F) Reactome, and the (G) Bioplanet.

70 common DEGs were enriched in 334 terms, including 253 biological processes, 65 molecular functions, and 16 cellular components (S4 Table). Then we summarized the top 10 terms according to P-value in each category in Table 2 and visualized in Fig 3A–3C. It can be found that many of these terms are related to metabolism and immunity, such as lipid biosynthetic process (GO:0008610) and negative regulation of dendritic cell apoptotic process (GO:2000669), which have a strong association with COVID-19 and ICC.

**Table 2:** Ontological analysis of common DEGs between COVID-19 and ICC.

Category	GO ID	Term	P-value	Genes
Biological Process	GO:0008610	lipid biosynthetic process	1.78E-04	*XBP1/SCD/CYP51A1/ACSL5*
	GO:2000669	negative regulation of dendritic cell apoptotic process	2.51E-04	*CCL21/LILRB1*
	GO:0048247	lymphocyte chemotaxis	4.91E-04	*CCL21/CCL20/CYP7B1*
	GO:2000668	regulation of dendritic cell apoptotic process	6.51E-04	*CCL21/LILRB1*
	GO:1901617	organic hydroxy compound biosynthetic process	7.15E-04	*PSAT1/HSD17B4/CYP7B1*
	GO:2000107	negative regulation of leukocyte apoptotic process	7.79E-04	*CCL21/LILRB1*
	GO:0043547	positive regulation of GTPase activity	9.21E-04	*ITGB1/ARHGAP11A/CCL21/GRTP1/CCL20*
	GO:0010560	positive regulation of glycoprotein biosynthetic process	1.07E-03	*CCL21/SLC2A10*
	GO:2000403	positive regulation of lymphocyte migration	1.07E-03	*CCL21/CCL20*
	GO:0072330	monocarboxylic acid biosynthetic process	1.40E-03	*XBP1/HSD17B4/CYP7B1*
Cellular Component	GO:1903561	extracellular vesicle	1.16E-03	*FGB/GPM6A/PROM1*
	GO:0098858	actin-based cell projection	3.09E-03	*ITGB1/GPM6A/PROM1*
	GO:0065010	extracellular membrane-bounded organelle	1.65E-02	*FGB/GPM6A*
	GO:0030868	smooth endoplasmic reticulum membrane	1.74E-02	*FTCD*
	GO:0030175	filopodium	1.76E-02	*ITGB1/GPM6A*
	GO:0031093	platelet alpha granule lumen	2.31E-02	*FGB/ALDOA*
	GO:0005759	mitochondrial matrix	3.37E-02	*AMT/NAGS/ACSS1/ACADSB*
	GO:0042627	chylomicron	3.45E-02	*APOC1*
	GO:0005789	endoplasmic reticulum membrane	3.82E-02	*SCD/CYP51A1/ACSL5/CHPT1/CYP7B1/FTCD*
	GO:0031091	platelet alpha granule	3.97E-02	*FGB/ALDOA*
Molecular Function	GO:0008168	methyltransferase activity	7.15E-04	*AS3MT/AMT/LCMT1*
	GO:0008376	acetylgalactosaminyltransferase activity	4.01E-03	*B3GNT3/B4GALNT4*
	GO:0004175	endopeptidase activity	4.93E-03	*F9/KDM8/MMP28/CASP2/HABP2*
	GO:0008395	steroid hydroxylase activity	7.04E-03	*CYP2A7/CYP7B1*
	GO:0048020	CCR chemokine receptor binding	9.50E-03	*CCL21/CCL20*
	GO:0008757	S-adenosylmethionine-dependent methyltransferase activity	9.94E-03	*AS3MT/LCMT1*
	GO:0008009	chemokine activity	1.13E-02	*CCL21/CCL20*
	GO:0042379	chemokine receptor binding	1.33E-02	*CCL21/CCL20*
	GO:0015485	cholesterol binding	1.33E-02	*APOF/PROM1*
	GO:0098639	collagen binding involved in cell-matrix adhesion	1.74E-02	*ITGB1*

We found 22 reliable pathways in Kyoto Encyclopedia of Genes and Genomes (KEGG), 32 reliable pathways in Wikipathways, 62 reliable pathways in Reatcome, and 77 reliable pathways in Bioplanet (S5 Table). The top 10 reliable pathways found in each database are listed in Table 3, and the bar graphs of pathway enrichment analysis are shown in Fig 3D–3G. In these pathways, more about the metabolic pathways were discovered, such as glyoxylate and dicarboxylate metabolism in KEGG, fatty acid biosynthesis in Wikipathways, metabolism of steroids in Reactome, and bile acid and bile salt metabolism in BioPlanet, which indicated that COVID-19 and ICC have common effects on these pathways.

**Table 3:** Pathway enrichment analysis of common DEGs between COVID-19 and ICC.

Category	Pathway	P-value	Genes
KEGG	Glyoxylate and dicarboxylate metabolism	1.56E-04	*AMT/ACSS1/ACAT2*
	Fatty acid degradation	4.58E-04	*ACSL5/ACADSB/ACAT2*
	Primary bile acid biosynthesis	1.59E-03	*HSD17B4/CYP7B1*
	Glycolysis / Gluconeogenesis	1.68E-03	*HKDC1/ALDOA/ACSS1*
	One carbon pool by folate	2.20E-03	*AMT/FTCD*
	Complement and coagulation cascades	3.30E-03	*FGB/C8G/F9*
	Biosynthesis of unsaturated fatty acids	4.01E-03	*SCD/HSD17B4*
	Fructose and mannose metabolism	5.94E-03	*HKDC1/ALDOA*
	Propanoate metabolism	6.30E-03	*ACSS1/ACAT2*
	Various types of N-glycan biosynthesis	8.23E-03	*MAN1A1/B4GALNT4*
Wikipathway	Complement and Coagulation Cascades	1.10E-03	*FGB/C8G/F9*
	Oxidation by Cytochrome P450	1.28E-03	*CYP2A7/CYP51A1/CYP7B1*
	Sterol regulatory element-binding proteins (SREBP) signaling	1.82E-03	*SCD/CYP51A1/ACSS1*
	Fatty Acid Biosynthesis	2.67E-03	*SCD/ACSL5*
	Blood Clotting Cascade	2.91E-03	*FGB/F9*
	Unfolded protein response	3.17E-03	*XBP1/CASP2*
	Statin inhibition of cholesterol production	4.61E-03	*APOC1/ACSS1*
	Gastric Cancer Network 1	4.61E-03	*ESM1/RUVBL1*
	One-carbon metabolism	4.93E-03	*AMT/FTCD*
	Nuclear Receptors Meta-Pathway	5.20E-03	*SLCO1B1/SLC2A10/CCL20/SCD/CES3*
Reactome	Phase I—Functionalization Of Compounds	1.71E-06	*CYP2A7/CYP51A1/CYP7B1/ACSS1/CES3/BPHL*
	Biological Oxidations	1.11E-05	*CYP2A7/AS3MT/CYP51A1/CYP7B1/ACSS1/CES3/BPHL*
	Metabolism	5.46E-05	*CYP51A1/AMT/ACSL5/HSD17B4/CYP7B1/BPHL/ACADSB/FTCD/CYP2A7/SLCO1B1/AS3MT/PSAT1/SCD/B3GNT3/UPP2/CHPT1/NAGS/ACSS1/CES3*
	Metabolism Of Steroids	1.99E-04	*SLCO1B1/SCD/CYP51A1/HSD17B4/CYP7B1*
	Bile Acid And Bile Salt Metabolism	5.24E-04	*SLCO1B1/HSD17B4/CYP7B1*
	Cytochrome P450—Arranged By Substrate Type	1.54E-03	*CYP2A7/CYP51A1/CYP7B1*
	Plasma Lipoprotein Assembly, Remodeling, And Clearance	1.98E-03	*APOC1/APOF/CES3*
	Synthesis Of Bile Acids And Bile Salts Via 7Alpha-Hydroxycholesterol	3.17E-03	*HSD17B4/CYP7B1*
	Endogenous Sterols	4.01E-03	*CYP51A1/CYP7B1*
	Synthesis Of Bile Acids And Bile Salts	6.30E-03	*HSD17B4/CYP7B1*
BioPlanet	Metabolism	3.08E-05	*CYP51A1/AMT/ACSL5/HSD17B4/CYP7B1/ACADSB/FTCD/ACAT2/CYP2A7/PSAT1/B3GNT3/MAN1A1/UPP2/CHPT1/NAGS/ALDOA/ACSS1*
	Bile acid and bile salt metabolism	1.13E-04	*SLCO1B1/HSD17B4/CYP7B1*
	Fatty acid metabolism	4.27E-04	*ACSL5/ACADSB/ACAT2*
	Cytochrome P450 metabolism of endogenous sterols	1.23E-03	*CYP51A1/CYP7B1*
	Extrinsic prothrombin activation pathway	1.23E-03	*FGB/F9*
	Cytochrome P450 pathway	1.28E-03	*CYP2A7/CYP51A1/CYP7B1*
	Primary bile acid biosynthesis	1.40E-03	*HSD17B4/CYP7B1*
	Phase I of biological oxidations: functionalization of compounds	1.82E-03	*CYP51A1/CYP7B1/ACSS1*
	Complement and coagulation cascades	1.90E-03	*FGB/C8G/F9*
	Blood clotting cascade	2.91E-03	*FGB/F9*

### 3.3 Protein-protein interaction networks analysis and identification of hub genes

**Fig 4:**
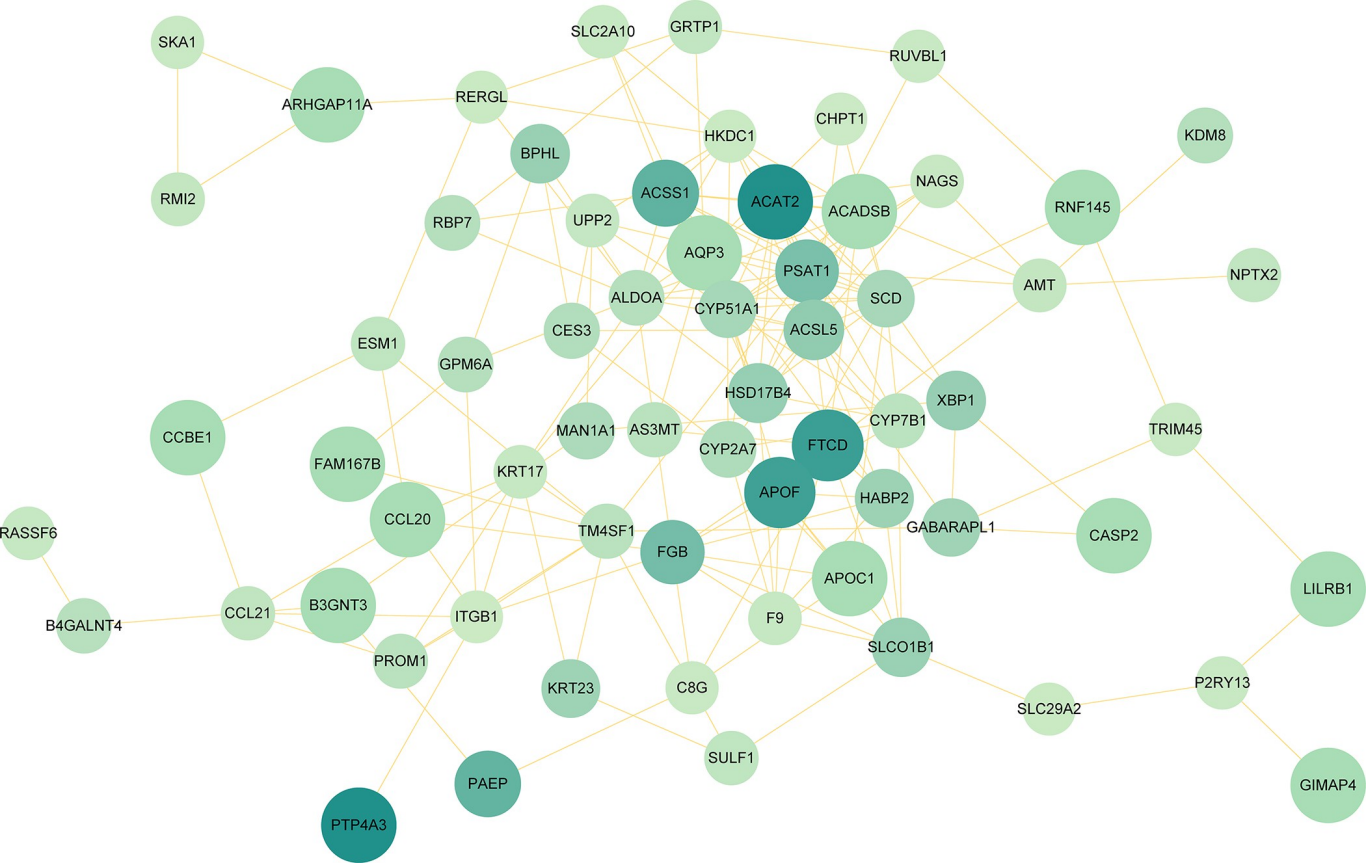
PPI network of common DEGs between COVID-19 and ICC. The circular nodes in the figure stand in for DEGs, while the edges indicate node interactions. The PPI network consists of 177 edges and 65 nodes. String was used to create the PPI network, and Cytoscape was used to display it.

**Fig 5:**
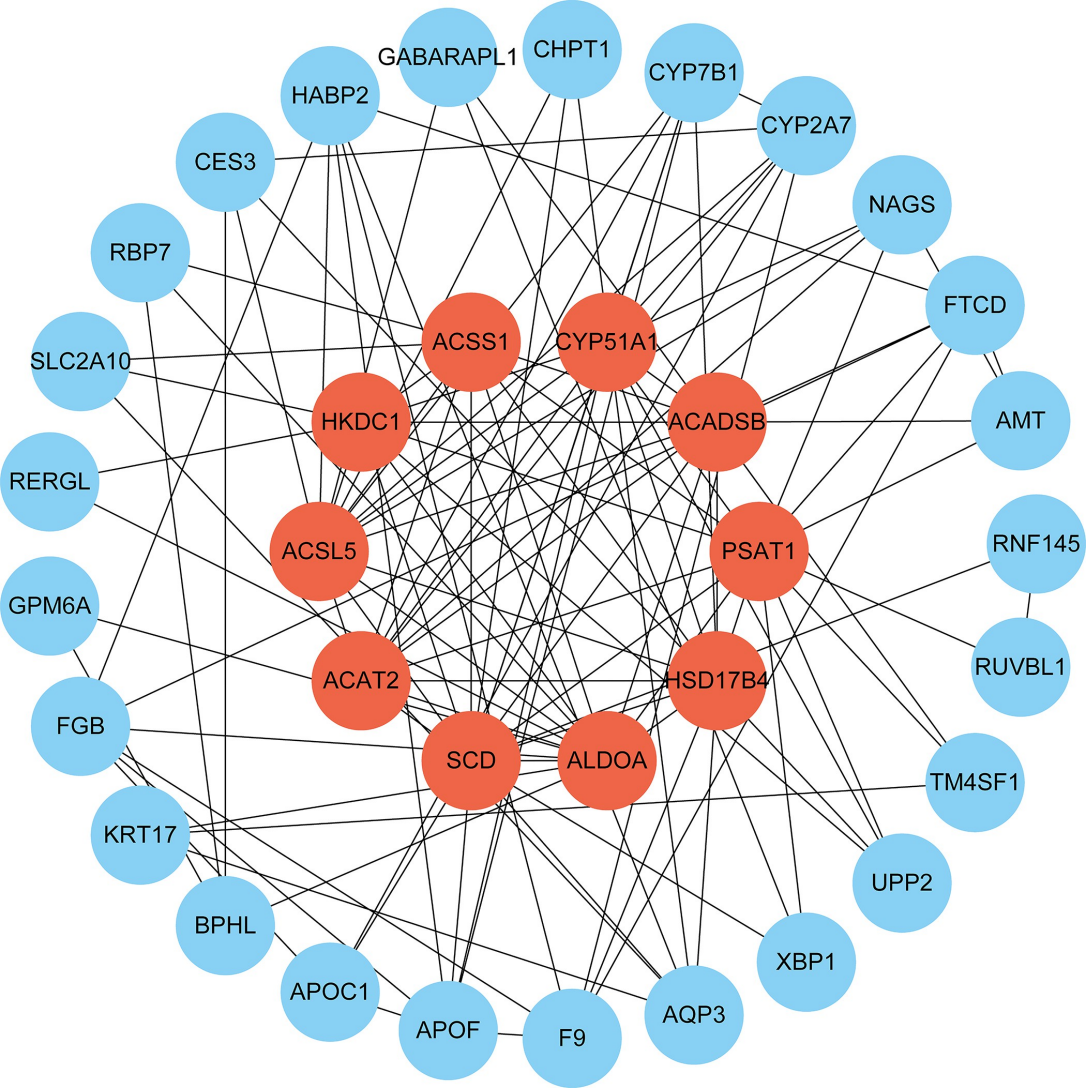
Determination of hub genes from the PPI network by using the Cytohubba plugin in Cytoscape. Hub genes were obtained using the Cytohubba plugin. Here, the red nodes indicate the highlighted top 10 hub genes and their interactions with other molecules. The network consists of 35 nodes and 144 edges.

To better understand biological signals, response mechanisms of energy substance metabolism, and functional links between proteins in disease states, this study obtained the PPI network via STRING. Subsequently, PPI was visualized in Cytoscape to forecast interaction between common protein-coding DEGs. The PPI network of common DEGs consists of 65 nodes and 177 edges (Fig 4 and S6 Table). According to PPI network analysis integrating Cytohubba plugin in Cytoscape, we ranked the most interconnected nodes top 10 DEGs (14.28%) as hub genes. The hub genes are as follows: *SCD*, *ACSL5*, *ACAT2*, *HSD17B4*, *ALDOA*, *ACSS1*, *ACADSB*, *CYP51A1*, *PSAT1*, and *HKDC1*. With the aid of the Cytohubba plugin, we also built a network of submodules to better comprehend their closeness and close connection, including 35 nodes and 114 edges (Fig 5). In the following analysis, we will focus on these 10 hub genes. These hub genes show potential biomarkers that can provide new therapeutic strategies for COVID-19 and ICC.

### 3.4 Construction of regulatory networks at transcriptional level

**Fig 6:**
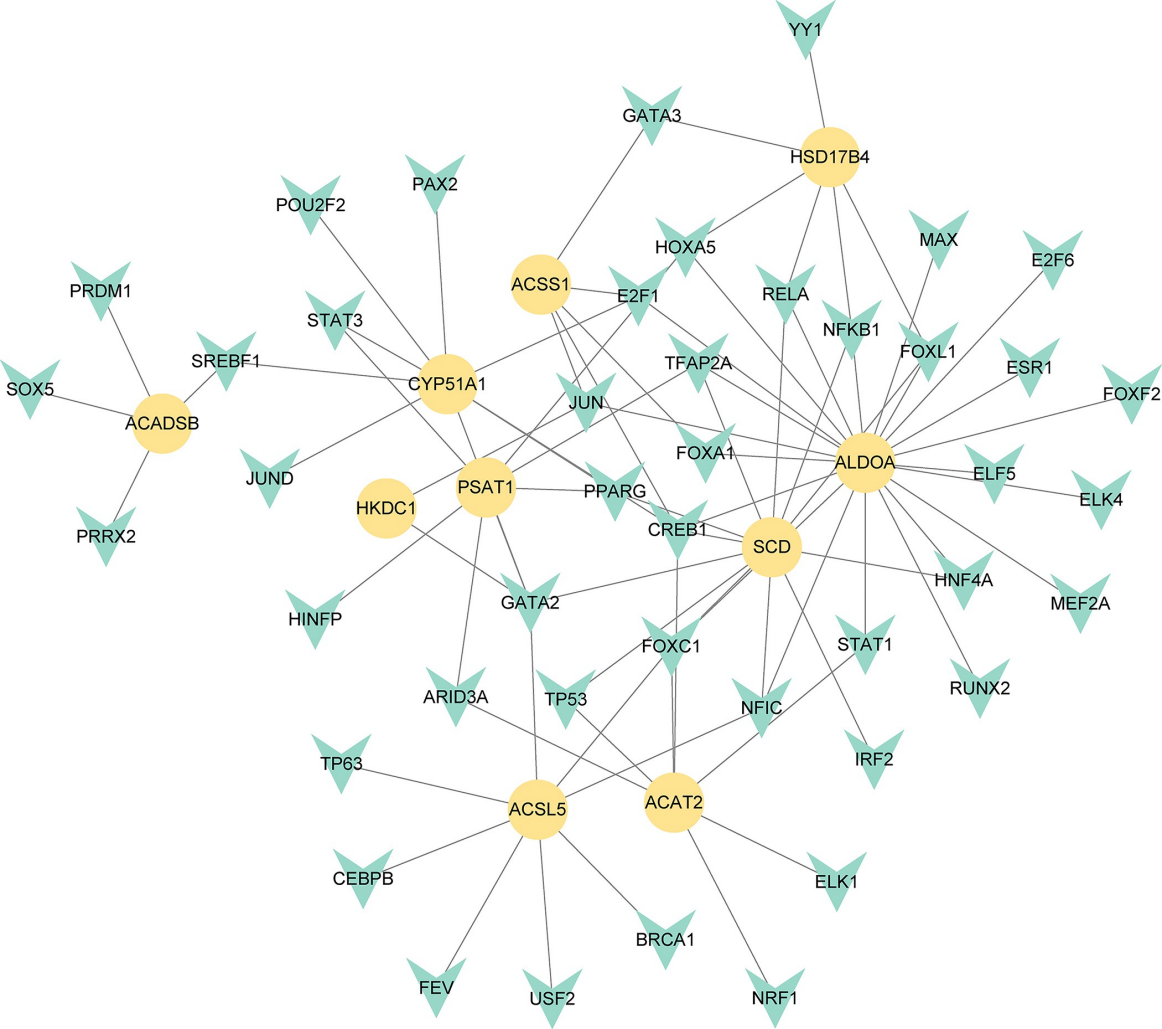
Interaction network of hub-gene-TFs. The cohesive regulatory interaction network of hub-gene-TFs obtained from the Network Analyst and described by Cytoscape. Herein, the green nodes are TFs, and the yellow nodes are hub genes.

To better understand the regulatory hub genes and detect the key alterations at the transcriptional level, we used network analysis to search for TFs and miRNAs of regulatory hub genes. The TFs-hub genes interactions are shown in Fig 6, and the information of interaction is presented in S7 Table. In the network, 44 TFs have been found. And ALDOA, CYP51A1, ACSL5, SCD, and CREB1 were more highly expressed among hub genes as genes have a higher degree in the network of TF-hub gene interactions. S1 Fig and S8 Table depict the relationships of miRNA-hub genes. By the similar method, multiple discovered hub genes were projected to be regulated by 112 miRNAs, such as *SCD*, *ALDOA*, *PSAT1*, *and CYP51A1*. In-depth study of these genes has common implications for treating COVID-19 and ICC.

### 3.5 Gene-disease association analysis

**Fig 7:**
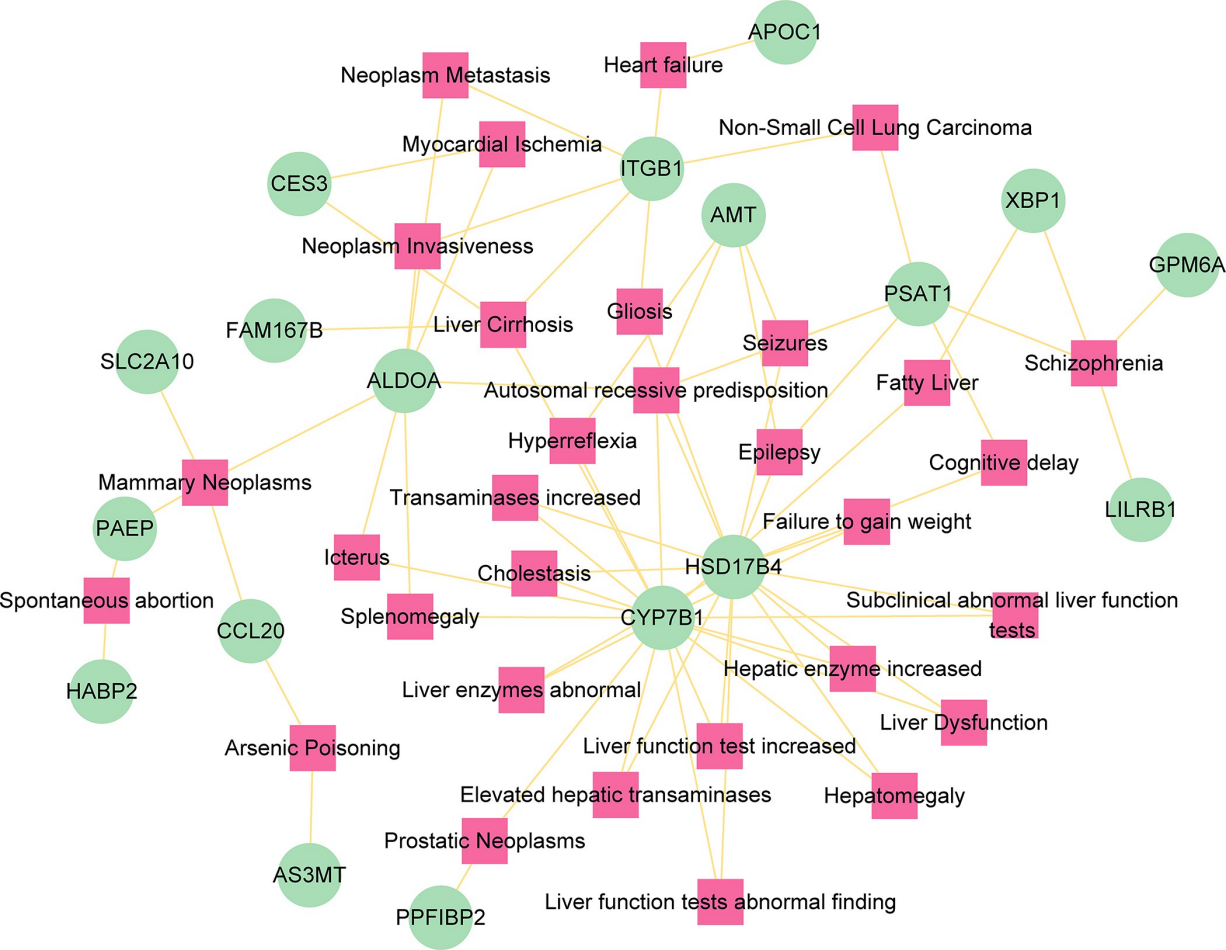
The gene-disease association network represents diseases associated with common genes. The diseases are depicted by the square node and gene symbols are defined by the circle node.

If different diseases have one or more similar genes, then we consider these diseases to be related to each other [34]. A total of 263 diseases were found to be associated with common genes and screened for significant diseases associated with at least two common genes (Fig 7). In our network, many diseases related to liver and cancer have been found, such as cholestasis, elevated hepatic transaminases, fatty liver, liver cirrhosis, liver dysfunction, mammary neoplasms, neoplasm invasiveness, neoplasm metastasis, non-small cell lung carcinoma, and prostatic neoplasms. Besides, the gene-disease association analysis also found some psychiatric disorders, including epilepsy, hyperreflexia, schizophrenia, and cognitive delay. These results portend the common association of COVID-19 and ICC with these diseases.

### 3.6 Identification of candidate drugs

To discover potential drugs for COVID-19 and ICC, we analyzed the protein-drug interactions of hub genes [35]. Potential therapeutic drugs were identified from the DSigDB database based on transcription characteristics using enrichment, and the top 10 candidate compounds (Periodate-oxidized adenosine, Desipramine, Quercetin, Perfluoroheptanoic acid, Tetrandrine, Pentadecafluorooctanoic acid, Benzo[a]pyrene, SARIN, Dorzolamide, 8-Bromo-cAMP) were identified based on P-value. The top ten enriched drugs in the DSigDB database are shown in Table 4, which indicated potential therapeutic effects on both COVID-19 and ICC.

**Table 4:** The recommended drugs for COVID-19 and ICC.

Name	P-value	Chemical Formula
Periodate-oxidized adenosine CTD 00001296	2.69E-06	C_10_H_11_N_5_O_4_
desipramine PC3 UP	4.63E-06	C_18_H_22_N_2_
quercetin CTD 00006679	1.28E-05	C_15_H_10_O_7_
Perfluoroheptanoic acid CTD 00003374	6.71E-05	C_7_HF_13_O_2_
tetrandrine MCF7 UP	7.27E-05	C_38_H_42_N_2_O_6_
Pentadecafluorooctanoic acid CTD 00001078	1.19E-04	C_8_HF_15_O_2_
benzo[a]pyrene CTD 00005488	1.66E-04	C_20_H_12_
SARIN CTD 00006722	1.70E-04	C_4_H_10_FO_2_P
dorzolamide HL60 DOWN	1.88E-04	C_10_H_16_N_2_O_4_S_3_
8-Bromo-cAMP, Na CTD 00007044	2.02E-04	C_10_H_10_BrN_5_NaO_6_P

## 4. Discussion

The 2019 SARS-CoV-2 global pandemic has riveted the world’s attention. With more and more variants of the virus, the transmission rate and morbidity rate of COVID-19 gradually increased. Although COVID‐19 primarily affects the respiratory system, liver dysfunction is also common in COVID-19 patients, such as elevated liver transaminases and elevations of cholestatic liver enzymes [36]. ICC, which is the second most common liver cancer, may be linked to COVID-19. To verify this idea, we use bioinformatics methods to find the relationship between these two diseases, and dig out some potential drugs This study could establish a link between COVID-19 and ICC and suggest possible treatment options for ICC patients infected with COVID-19.

Lipid biosynthetic process (GO:0008610), glyoxylate and dicarboxylate metabolism pathway (KO:00630), and fatty acid degradation pathway (KO:00071) are found in GO terms and KEGG pathways. Research detected that COVID-19 patients was dysregulated metabolites involved in lipid metabolism [37]. Another study also found that SARS-CoV-2 infection elevated the expression of the RE1-silencing transcription factor (REST), which regulated the transcriptional expression of secreted metabolic factors such as myeloperoxidase, apelin, and myostatin, causing disruptions in glucose and lipid metabolism [38]. Moreover, recent studies have found that altered lipid metabolism is a new hallmark of cancer [39]. A study found that KDM5C, a histone H3K4-specific demethylase, can repress FASN-mediated lipid metabolism to exert tumor suppressor activity in ICC [40]. Consistent with the results of GO and pathway analysis, there are also many genes related to metabolism in hub genes. Stearoyl-CoA desaturase (*SCD*) was reported to plays a key role in lipid biosynthesis pathways involved in tumorigenesis, and so pharmacological inhibitors have been developed such as MF-438, CAY10566 and A939572 [41], but has few research in ICC. In addition, both *ACSL5* and *HSD17B4* were found to be associated with fatty acid synthesis, which may indicate the impact of COVID-19 and ICC on lipid metabolism. These results suggested that COVID-19 and ICC may jointly affect lipid metabolic function of human body. However, whether lipid metabolism can be a therapeutic target for these two diseases needs further study.

Regulation of dendritic cell apoptotic process (GO:2000668), negative regulation of leukocyte apoptotic process (GO:2000107), positive regulation of GTPase activity (GO:0043547), and positive regulation of lymphocyte migration (GO:2000403) are related to immunity, which suggested that both ICC and COVID-19 have a huge impact on the immune system. SARS-CoV-2 has been demonstrated to alter normal immune responses, resulting in a weakened immune system and uncontrolled inflammatory reactions in COVID-19 severe and critical patients [42], which is the major cause of ARDS. Plasma levels of IL-2, IL-7, IL-10, granulocyte colony-stimulating factor (G-CSF), IP-10, MCP1, macrophage inflammatory protein 1α (MIP1α), and tumor necrosis factor (TNF) have been observed in patients with severe COVID-19 were higher than in healthy adults [43]. On the other hand, cancer is usually associated with immune escape by suppressing the immune system. A study found that tumor-derived exosomal miR-183-5p up-regulates PD-L1-expressing macrophages to foster immune suppression and disease progression in ICC through the miR-183-5p/PTEN/AKT/PD-L1 pathway [44]. Additionally, in this study, complement and coagulation cascades related pathways are found in top 10 pathway in each database. It has been shown that SARS-CoV-2 may activate the complement system’s classical and lectin pathways [45], and lectin pathways components were found deposited in lung tissue of COVID-19 patients [46], which is consistent with the results of our pathway analysis. Meanwhile, the complement system may be involved in liver dysfunction in viral-induced acute liver failure cases [47]. The aforementioned hub protein SCD also plays a role in immune function. A recent study found that suppression of SCD reduces humoral immune response to immunization and weakens immune defense against respiratory influenza infection [48]. But SCD1 expressed in cancer cells and immune cells causes immune resistance conditions, and its inhibition augments antitumor T cells and therapeutic effects of anti-PD-1 antibody [49]. Not only that, hub gene PSAT1 can also enhance immunosuppressive through PERK-ATF4-PSAT1 axis in tumor [50, 51]. CREB1, a TF with the highest correlation score in our TF-hub gene interaction analysis, was reported to promote T cell cytotoxicity [52]. In conclusion, both COVID-19 and ICC can elicit immune system responses. COVID-19 usually causes elevated inflammatory immune response, while ICC causes immune suppression. But the combined effect of these two immune responses on human body is unknown.

There was a report that a patient diagnosed with advanced Hodgkin’s lymphoma, who was not being treated for lymphoma, contracted COVID-19 and four months after ending treatment for COVID-19, was re-examined by PET-CT and found that most of his tumors had disappeared, with levels of biomarkers associated with the tumor dropping by more than 90% [53]. Interestingly, the associations between COVID-19 and cancer were also identified in our study. The hub gene in our analysis *ACSL5*, *ALDOA*, and *HKDC1* are directly associated with liver cancer [54–56]. Besides, gene-disease network analysis found some cancer related diseases, including mammary neoplasms, non-small cell lung carcinoma, and prostatic neoplasms. At the same time, neoplasm invasiveness and neoplasm metastasis are also showing in the result, which suggested that the ICC patient with COVID-19 may have a risk of developing other types of tumors and metastases. Similarly, in TF-gene network, SREBF1 was found to enhance the viability and motility in cancer [57]. The above evidences suggest that COVID-19 may have an effect on tumor migration and metastasis in ICC patients, but the detailed effect and mechanism require further investigation.

Regarding drug prediction, several chemical substances have shown promise as potential treatments for COVID-19, including quercetin and tetrandrine [58, 59]. Notably, these drugs also possess anti-cancer properties. Quercetin can influence pathways such as PI3K/Akt/mTOR, Wnt/β-catenin, and MAPK/ERK1/2 to induce apoptosis in cancer cells [60]. Tetrandrine, another candidate, has anti-angiogenic properties [61]. Therefore, it is plausible that ICC patients infected with COVID-19 could benefit from these drugs. Besides, the top 2 candidates, Periodate-oxidized adenosine and desipramine, have been reported to have some anticancer effects [62, 63], despite not being originally intended for that purpose.

However, bioinformatics, which is based on the advancement of modern computer technology and the simplicity of biological experimental techniques, cannot replace clinical testing [64]. Additionally, the selected datasets in this study include different groups of people with two different diseases, rather than the same population with both ICC and COVID-19, which may lead to some differences between the results of our analysis and the actual results. To ensure the credibility of these findings, it is essential to conduct *in vivo*, in vitro, and clinical studies to validate the results of the bioinformatics analysis. Furthermore, this study suggests that therapeutic approaches for ICC and COVID-19 comorbidity can be further explored from the perspective of lipid metabolism and immunology. And whether potential candidate drugs can treat ICC and covid-19 at the same time is also a topic worthy of further research.

## 5. Conclusions

To help gain insight into the connection between ICC and COVID-19, we utilized transcriptomic data analysis to identify differentially expressed genes shared in both diseases. A total of 70 common DEGs and 10 hub genes revealed certain similarities between ICC and COVID-19 in terms of pathogenic processes. Further, we identified 44 TFs and 112 miRNAs by building a transcriptional regulatory network targeting hub genes. Notably, drug prediction results indicate quercetin and tetrandrine as potential agents for the treatment of ICC and COVID-19. Although our study has certain limitations, these results can provide ideas and directions for subsequent research on the two diseases, such as target screening, targeted therapy, and drug development. Overall, this study could shed new light on the treatment and drug development of ICC and COVID-19.

## Supporting information

S1 Table Differentially expressed genes in GSE119336. (XLSX)

S2 Table Differentially expressed genes in GSE152418. (XLSX)

S3 Table Shared DEGs between GSE119336 and GSE152418. (XLSX)

S4 Table GO terms of common DEGs. (XLSX)

S5 Table KEGG, Wikipathways, Reactome, and Bioplanet of common DEGs. (XLSX)

S6 Table PPI network of common DEGs. (XLSX)

S7 Table TF-Gene topology table. (XLSX)

S8 Table miRNA-Gene topology table. (XLSX)

S1 Fig The interconnected regulatory interaction network of hub-gene-miRNAs. Herein, the blue nodes indicate miRNAs and the red nodes are hub genes.
(TIF)
