# Supplementary figures and images for: Bioinformatics and system biology approaches to determine the connection of SARS-CoV-2 infection and intrahepatic cholangiocarcinoma
Source: PLoS One. 2024 Apr 22;19(4):e0300441. doi: 10.1371/journal.pone.0300441 (PMC11034673; doi:10.1371/journal.pone.0300441)

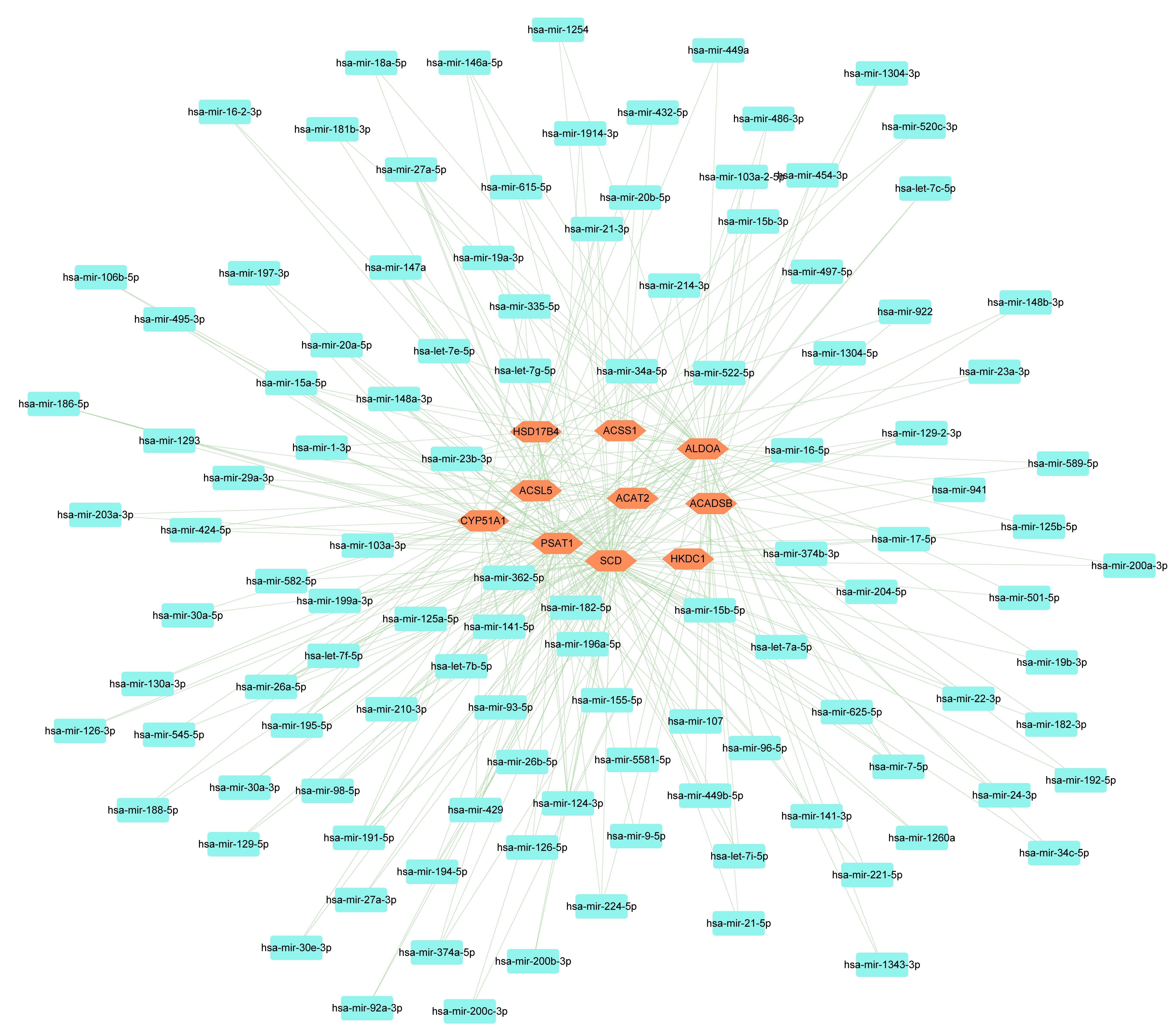

Supplement: S1 Fig — Herein, the blue nodes indicate miRNAs and the red nodes are hub genes. (TIF) [file pone.0300441.s009.tif]
